# The Three Flagellar Loci of *Brucella ovis* PA Are Dispensable for Virulence in Cellular Models and Mice

**DOI:** 10.3389/fvets.2020.00441

**Published:** 2020-07-31

**Authors:** Rebeca S. Sidhu-Muñoz, Carmen Tejedor, Nieves Vizcaíno

**Affiliations:** ^1^Departamento de Microbiología y Genética, Universidad de Salamanca, Salamanca, Spain; ^2^Instituto de Investigación Biomédica de Salamanca (IBSAL), Salamanca, Spain

**Keywords:** *Brucella ovis*, virulence, flagella, deletion mutant, intracellular survival, mouse model

## Abstract

*Brucella ovis* is a facultative intracellular bacterium that causes a non-zoonotic ovine brucellosis mainly characterized by male genital lesions and is responsible for important economic losses in sheep farming areas. Studies about the virulence mechanisms of *Brucella* have been mostly performed with smooth (bearing O-polysaccharide in lipopolysaccharide) zoonotic species, and those performed with *B. ovis* have revealed similarities but also relevant differences. Except for few strains recently isolated from unconventional hosts, *Brucella* species are non-motile but contain the genes required to assemble a flagellum, which are organized in three main loci of about 18.5, 6.4, and 7.8 kb. Although these loci contain different pseudogenes depending on the non-motile *Brucella* species, smooth *B. melitensis* 16M builds a sheathed flagellum under particular culture conditions and requires flagellar genes for virulence. However, nothing is known in this respect regarding other *Brucella* strains. In this work, we have constructed a panel of *B. ovis* PA mutants defective in one, two or the three flagellar loci in order to assess their role in virulence of this rough (lacking O-polysaccharide) *Brucella* species. No relevant differences in growth, outer membrane-related properties or intracellular behavior in cellular models were observed between flagellar mutants and the parental strain, which is in accordance with previous results with *B. melitensis* 16M single-gene mutants. However, contrary to these *B. melitensis* mutants, unable to establish a chronic infection in mice, removal of the three flagellar loci in *B. ovis* did not affect virulence in the mouse model. These results evidence new relevant differences between *B. ovis* and *B. melitensis*, two species highly homologous at the DNA level and that cause ovine brucellosis, but that exhibit differences in the zoonotic potential, pathogenicity and tissue tropism.

## Introduction

The genus *Brucella* is constituted by six classical species (*B. melitensis, B. abortus, B. suis, B. canis, B. ovis*, and *B. neotomae*), that cause brucellosis in terrestrial mammals, and six other species (*B. ceti, B. pinnipedialis, B. microti, B. inopinata, B. vulpis*, and *B. papionis*) that have been isolated since the 1990s from other terrestrial mammals or from marine mammals (https://lpsn.dsmz.de/genus/brucella). The *Brucella* spp. host range has more recently increased to amphibians and fish, with atypical strains isolated from several frog species and a ray (1, 2). Despite the high percentage of DNA-DNA hybridization detected among the classical *Brucella* species (96 ± 5% when compared to *B. melitensis* 16M) (3), some differential genetic markers have been found and they differ in several phenotypic characteristics, host preference and pathogenicity. Nevertheless, a common trait is their ability to survive and replicate inside phagocytic cells (4–7). The *Brucella* species are smooth (S) or rough (R) depending on the presence or absence, respectively, of O-polysaccharide chains in the lipopolysaccharide (LPS). *B. ovis* and *B. canis* are the only rough *Brucella* species but are virulent for their natural hosts (sheep and dogs, respectively), which contrasts with the other *Brucella* species that are smooth and require S-LPS for full virulence (8–10).

Although studies regarding the virulence of R strains has increased in the last years, most work in this respect has been performed with S *Brucella* species (mainly with zoonotic *B. melitensis, B. abortus*, and *B. suis*). Among the genes involved in the virulence of smooth *B. melitensis*, flagellar genes are required for the establishment of a chronic infection in mice (11), which constitutes an intriguing trait since *B. melitensis* is a non-motile species (1). In fact, among the brucellae only *B. inopinata* and the *Brucella* atypical strains isolated from frogs and a ray are motile (1, 2, 12–14) and at least frog isolates are able to build a polar flagellum in culture medium (1). Despite the presence of several pseudogenes in the three main flagellar loci (1, 11) and its non-motile character (1), *B. melitensis* 16M is able to build a sheathed polar flagellum in the early exponential phase of growth (11) and, as mentioned above, flagellar mutants of *B. melitensis* 16M are attenuated in virulence (11). Although the three flagellar loci are conserved in the genus *Brucella*, with a different pattern of pseudogenization in most cases, no additional studies have been performed to evaluate the relevance of flagellar genes in the virulence other *Brucella* species. According to its rough phenotype, its particular outer membrane (OM)-related and virulence characteristics and its shared preference with *B. melitensis* by the ovine host (15–21), we have selected *B. ovis* to extend the knowledge about the role of flagellar genes in the virulence of the genus *Brucella*. With this aim, we have constructed a panel of flagellar mutants in rough virulent *B. ovis* PA (with one, two or the three flagellar loci deleted) that has been characterized regarding growth characteristics, OM-related properties, intracellular behavior in cellular models of professional and non-professional phagocytes and virulence in the mouse model.

## Materials and Methods

### Plasmids, Bacterial Strains, and Culture Conditions

Plasmids pGEM-T Easy (Promega, Madison, WI, United States) and pCVDKan-D (18) were used to construct the recombinant plasmids containing the inactivated flagellar loci. They were maintained in *Escherichia coli* JM109 and CC118 (λpir), respectively. Recombinant *E. coli* strains were cultured at 37°C in Luria Bertani (LB) medium supplemented with 50 μg/ml ampicillin (pGEM-T Easy derived plasmids) or kanamycin (pCVDKan-D derived plasmids).

Virulent *B. ovis* PA was used as parental strain to obtain the panel of flagellar mutants and as reference strain for comparisons in the different assays. It was obtained from the bacterial culture collection maintained at the Institut National de la Recherche Agronomique, Nouzilly, France. *B. ovis* strains were cultured in tryptic soy agar (TSA) or tryptic soy broth (TSB) (Pronadisa-Laboratorios Conda, Torrejón de Ardoz, Spain), supplemented with 0.3% yeast extract (YE) (Pronadisa-Laboratorios Conda, Torrejón de Ardoz, Spain) and 5% horse serum (HS) (Gibco-Life Technologies, Grand Island, NY, United States). When required for the mutagenesis procedure, TSA-YE-HS was supplemented with kanamycin (Kan) at a final concentration of 50 μg/ml or with 5% sucrose (Sigma-Aldrich, St. Louis, MO, United States). *B. ovis* strains were cultured at 37°C under a 5% CO_2_ atmosphere.

### *In silico* DNA and Protein Analysis, Primers, and Nucleic Acid Techniques

Genomes of *B. melitensis* 16M (ATCC 23456) and *B. ovis* 63/290 (ATCC 25840) were analyzed from GeneBank data (accession numbers AE008917 and AE008918 for *B. melitensis* 16M chromosome I and II, respectively, and accession numbers NC_009505 and NC_009504 for *B. ovis* 63/290 chromosomes). Gene data for motile *Brucella* sp. B13-0095 isolated from a Pac-Man frog were retrieved from the Pathosystems Resource Integration Center (PATRIC; genome ID 1867845.3; https://www.patricbrc.org) (22). Orthologs were analyzed at the Kyoto Encyclopedia of Genes and Genomes (KEGG; https://www.kegg.jp) and protein and DNA alignments were performed with LALIGN (https://www.ebi.ac.uk/Tools/psa/lalign/) from the European Bioinformatics Institute (23). PSORTb v3.0.2 (Brinkman Laboratory, Simon Fraser University, British Columbia, Canada; https://www.psort.org/psortb/) was used to predict protein subcellular localization (24). Gene Construction Kit (GCK 4.5; Textco Biosoftware, Raleigh, NC, United States) was used as assistant tool for the analysis of nucleotide sequences and schematic drawing of the flagellar loci.

DNA primers (IDT, Leuven, Belgium) used for gene expression analysis and for the construction and characterization of mutant strains are described in Table 1. PCR amplification was performed with AccuPOL DNA polymerase (VWR, Leuven, Belgium), Red Taq DNA polymerase master mix (VWR, Leuven, Belgium) or Expand^TM^ Long Template PCR System (Roche, Mannheim, Germany), depending on the experiment. For gene expression studies, RNA was extracted with RNeasy mini kit (Qiagen, Hilden, Germany) from 5 × 10^9^ CFU of *B. ovis* that had been cultured in liquid medium for 16 or 49 h (t16 or t49; exponential and stationary growth phase, respectively). Residual DNA was removed with RQ1 DNase (Promega, Madison, WI, United States) and cDNA was synthetized with the first strand cDNA synthesis kit for RT-PCR (Roche, Mannheim, Germany) using the random hexamers provided with the kit as primers for reverse transcriptase (RT). Parallel control reactions were performed in the same conditions but omitting RT. Subsequent PCR reactions were performed (using the cDNA as template and a panel of primer pairs targeting genes in the three flagellar loci) either with the Expand^TM^ Long Template PCR System for end-point RT-PCR or with the KAPA SYBR® Fast Master Mix (Kapa Biosystems, Cape Town, South Africa) for relative quantification by real time RT-PCR (qRT-PCR). Four biological replicates, with three technical replicates each, were used in qRT-PCR assays that were performed in a StepOnePlus^TM^ device (Applied Biosystems, Foster City, CA, United States). Gene expression levels were determined, with the StepOne^TM^ software v2.3, by the 2^−ΔΔCt^ method with the *16S* gene as internal reference.

**Table 1:** Primers used in this work for the construction and verification of *B. ovis* PA flagellar mutants.

**Primer name**	**Nucleotide sequence 5'-3'^a^**	**Target locus or gene^b^**
**Construction of** ***B. ovis*** **PA flagellar mutants**		
Flg1MUT-F	AAATGCCCGGGATCATGT	Locus I
Flg1OVL-R	ATTGGCCTTGTTGTCGGA	Locus I
Flg1OVL-F	TCCGACAACAAGGCCAATGCCCGATGATCCGCATTA	Locus I
Flg1MUT-R	GATTCTGGCTCTTTGACG	Locus I
Flg2MUT-F	GCGGCAAGGCCATTTTCT	Locus II
Flg2OVL-R	CCTTGCAGCCAGATCGAA	Locus II
Flg2OVL-F	TTCGATCTGGCTGCAAGGGGCTGGAACATTCTGGTT	Locus II
Flg2MUT-R	TGCAAGCATGAGCGTCAA	Locus II
Flg3MUT-F2	GCTGCCAATGGCAAGACT	Locus III
Flg3OVL-R	CGCATCATCAACACACGG	Locus III
Flg3OVL-F	CCGTGTGTTGATGATGCGGACAGACAGGCGCAAAAC	Locus III
Flg3MUT-R	GGCGCGAGCTTGTATGTC	Locus III
**Additional primers for the verification of recombinant plasmids and mutants**		
Universal-F	GTTTTCCCAGTCACGAC	pGEM-T Easy
Universal-R	CAGGAAACAGCTATGAC	pGEM-T Easy
Flg1-F	AATGCTTCGTACTGGTCC	Locus I
Flg1-R	TCCCTTGAGCTGTTCGAT	Locus I
Flg2-F	TGAAGGGGCTCAATCAGA	Locus II
Flg2-R	GATCGCTTTGTTCATGCT	Locus II
Flg3-F	CCTATCCTTGGTTTCCGC	Locus III
Flg3-R	CGATGCAGGATGCAGTTG	Locus III
**Primers for RT-PCR or qRT-PCR**		
FliC RT-F	CAAACTCGTCGGCTCTGA	*fliC* (locus I)
Flg1OVL-R	ATTGGCCTTGTTGTCGGA	*fliC* (locus I)
FliF RT-F	TTGATGGGTGCGATCCTC	*fliF* (locus I)
FliF RT-R	CCTTGCCGATTGGAACGA	*fliF* (locus I)
FtcR RT-F	AGCCTTCCTGATTGGTGA	*ftcR* (locus I)
FtcR RT-R	ATTTCGCGGACATGAACG	*ftcR* (locus I)
FlgE RT-F	CGGAAACGCAATTCTCCT	*flgE* (locus I)
FlgE RT-R	TTGTCCGGCACGAAAGAA	*flgE* (locus I)
FlbT RT-F	CATCAATGGCGCGGTTCT	*flbT* (locus I)
FlbT RT-R	AACATGCCTTTCAGCATC	*flbT* (locus I)
FlgJ RT-F	AGGGCTGACGCAGGATAA	*flgJ* (locus I)
FlgJ RT-R	AAAGTCGCAGTCGTGTCG	*flgJ* (locus I)
FlgG RT-F	TGACGCTTGACGGCAATC	*flgG* (locus II)
FlgG RT-R	GTTCGAGACCGGCTTCAT	*flgG* (locus II)
FlhB RT-F	ATCGAAACCGGCAATGGC	*flhb* (locus III)
FlhB RT-R	CCGCAAGCGTCATCGTCT	*flhb* (locus III)
FlgF RT-F	GCTGATCAAGACCGACAA	*flgF* (locus III)
FlgF RT-R	GACATCGAGGATCGCATT	*flgF* (locus III)
16S-RT Fw	TCTCACGACACGAGCTGACG	*16S*
16S-RT Rv	CGCAGAACCTTACCAGCCCT	*16S*

a *Underlined sequences in Flg1OVL-F, Flg2OVL-F, and Flg3OVL-F2 correspond to regions overlapping with Flg1OVL-R, Flg2OVL-R, and Flg3OVL-R, respectively*.

b *Target gene is the B. ovis locus to be deleted or PCR-amplified for the verification of mutant strains or for RT-PCR. Primers Universal-F and Universal-R target pGEM-T Easy and its derived recombinant plasmids at both sides of the cloned insert and were used for sequencing of the DNA insert. The remaining primers target the B. ovis genome and were designed according to the published genome sequence of B. ovis 63/290 (ATCC 25840) (accession numbers NC_009505 and NC_009504 for chromosome I and II, respectively). Primers targeting 16S and fliF were those previously described (18, 25)*.

### Mutagenesis Procedure

Mutant strains for the three main flagellar loci (Table 2) were obtained by in-frame deletion with overlapping PCR as described previously (18). Briefly, for removal of the entire locus I, the 5′end and upstream DNA (about 700 bp) was PCR amplified with primers Flg1MUT-F and Flg1OVL-R and AccuPOL DNA polymerase. Similarly, the 3′ end and downstream DNA was amplified with primers Flg1OVL-F and Flg1MUT-R. Both fragments were fused, through the complementary regions of primers Flg1OVL-F and Flg1OVL-R (Table 1), with an overlapping PCR reaction with primers Flg1MUT-F and Flg1MUT-R and the Expand^TM^ Long Template PCR System. The resulting DNA fragment was ligated in pGEM-T Easy, verified by DNA sequencing, and then cloned in pCVDKan-D, a plasmid that confers resistance to kanamycin and sensibility to sucrose (18). The recombinant plasmid was introduced in parental *B. ovis* PA by electroporation. *B. ovis* PA colonies bearing the plasmid integrated in the chromosome, that consequently contains one copy of the wild type locus and one copy of the modified locus, were detected by plating on TSA-YE-HS plates containing kanamycin. Colonies were verified by PCR with appropriate primers to detect both copies of locus I (intermediate strain). Colonies suffering a second recombination event, leading either to the desired mutant strain or to a strain reverting to the wild type genotype, were detected by plating the intermediate strain on TSA-YE-HS plates containing sucrose. The differentiation between the mutant strain lacking flagellar locus I and the intermediate or wild type strains was performed by a series of PCR reactions with Red Taq DNA polymerase master mix and primers located inside and/or outside the deleted region. Mutants lacking the entire locus II or the entire locus III (Table 2) were obtained similarly with their specific primers (Table 1). The single mutants for each flagellar locus served as parental strains for a second round of mutation leading to the deletion of an additional flagellar locus. The double mutants obtained (Table 2) were subsequently used as parental strains to obtain the panel of triple mutants of *B. ovis* PA lacking the three main flagellar loci (Table 2).

**Table 2:** Most relevant *B. ovis* PA mutants in flagellar loci obtained in this work^a^.

***B. ovis* strain^b^**	**Deleted loci and order of deletion**	**bp deleted**
***B. ovis*** **PA single mutants (one entire locus deleted)**
**B. ovis Δ flg1**	Locus I completely deleted	18459
**B. ovis Δ flg2**	Locus II completely deleted	6320
**B. ovis Δ flg3**	Locus III completely deleted	7736
***B. ovis*** **PA double mutants (two entire loci deleted)**
*B. ovis* Δ*flg1*Δ*flg2*	Loci I and II completely deleted	24779
*B. ovis* Δ*flg1*Δ*flg3*	Loci I and III completely deleted	26195
*B. ovis* Δ*flg2*Δ*flg1*	Loci II and I completely deleted	24779
*B. ovis* Δ*flg2*Δ*flg3*	Loci II and III completely deleted	14056
*B. ovis* Δ*flg3*Δ*flg1*	Loci III and I completely deleted	26195
*B. ovis* Δ*flg3*Δ*flg2*	Loci III and II completely deleted	14056
***B. ovis*** **PA triple mutants (three entire loci deleted)**
**B. ovis Δ flg1 Δ flg2 Δ flg3**	Loci I, II, and III completely deleted	32515
*B. ovis* Δ*flg1*Δ*flg3*Δ*flg2*	Loci I, III, and II completely deleted	32515
**B. ovis Δ flg2 Δ flg1 Δ flg3**	Loci II, I, and III completely deleted	32515
*B. ovis* Δ*flg2*Δ*flg3*Δ*flg1*	Loci II, III, and I completely deleted	32515
*B. ovis* Δ*flg3*Δ*flg1*Δ*flg2*	Loci III, I, and II completely deleted	32515
**B. ovis Δ flg3 Δ flg2 Δ flg1**	Loci III, II, and I completely deleted	32515

a *Intermediate strains obtained during mutagenesis are not cited*.

b *Mutant strains phenotypically characterized in this work are highlighted in blue bold characters. Order of citation of the deleted loci in the strain name corresponds to the order of deletion of each locus (i.e., B. ovis Δflg2Δflg1 was obtained from parental B. ovis PA by deletion of locus II to obtain B. ovis Δflg2 and, then, by deletion of locus I from the Δflg2 single mutant)*.

### Growth, Autoagglutination, and Susceptibility Assays

Growth of mutant strains in solid and liquid medium was analyzed as previously described (26). Briefly, to evaluate growth in solid medium, bacterial suspensions in PBS with values of optical density at 600 nm (OD_600_) of 0.2 were appropriately diluted and plated on TSA-YE-HS plates to determine the numbers of CFU/ml after 5 days incubation. Growth curves in liquid TSB-YE-HS were also established by measuring the evolution of OD_600_ scores and log CFU/ml numbers of bacterial suspensions starting at OD_600_ values of 0.05 and incubated under agitation for 170 h.

To evaluate the autoagglutination ability, bacterial suspensions in TSB-YE-HS of OD_600_ values of 0.8 (100% OD_600_) were incubated for 48 h under static conditions to measure the evolution of the OD_600_ scores (18, 27). Susceptibility to polymyxin B, sodium deoxycholate and H_2_O_2_ (all from Sigma-Aldrich) was measured using a disc assay as follows. Bacterial suspensions (100 μl) with OD_600_ values of 0.2 were plated on TSA-YE-HS. Discs of 0.9-mm diameter (Los Productos de Aldo, Spain) were then placed in the middle of the plate and soaked with 20 μl of polymyxin B (250 000 UI/ml), sodium deoxycholate (10 mg/ml) or 30% H_2_O_2_. The diameter of the growth inhibition halo was recorded in quadruplicate for each plate after a 5-day incubation period and the results expressed as mean ± SD of three plates.

### Virulence Evaluation in Cellular Models and Mice

Intracellular behavior of mutant strains was studied in J774.A1 macrophages and HeLa cells as previously described (19). Briefly, 2 × 10^4^ J774.A1 macrophages/well or 1.5 × 10^4^ HeLa cells/well were cultured in 96-well plates for 24 h. Bacteria (4 × 10^6^ or 8 × 10^6^ CFU/well for J774.A1 or HeLa cells, respectively) were allowed to internalize for 2 h in the cell lines. Extracellular bacteria were killed with gentamycin and intracellular bacteria were enumerated in three wells per bacterial strain after lysis of the eukaryotic cells and plating on TSA-YE-HS (t0). The remaining wells were incubated in the presence of gentamycin to evaluate intracellular bacterial numbers at 20 and 44 h (t20 and t44) post-infection (p.i.).

Virulence in mice was evaluated in 6-week old female BALB/c mice (Charles River Laboratories, Chatillon-sur-Chalaronne, France) received 1 week before. They were intraperitoneally inoculated with 10^6^ CFU of parental *B. ovis* PA or the flagellar triple mutants *B. ovis* Δ*flg1*Δ*flg2*Δ*flg3, B. ovis* Δ*flg2*Δ*flg1*Δ*flg3* or *B. ovis* Δ*flg3*Δ*flg2*Δ*flg1*. Bacterial numbers in spleen were determined -as previously described (28)- in 5 mice per group at 3, 7, and 11 weeks p.i. (W3, W7, and W11), which in *B. ovis* PA corresponds to the peak of infection in the acute phase, to the chronic phase and to the decline phase of infection, respectively (26, 27).

### Statistical Analysis

Statistical comparisons were performed with one-way ANOVA and Fisher's Least Significant Differences test on a GraphPad Prims Software (GraphPad Software Inc., San Diego, CA, United States). Statistically significant differences (*P* < 0.01) were established with a 99% confidence interval.

## Results

### Genomic Organization and Transcription of the Flagellar Loci in *B. ovis*

**Figure 1:**
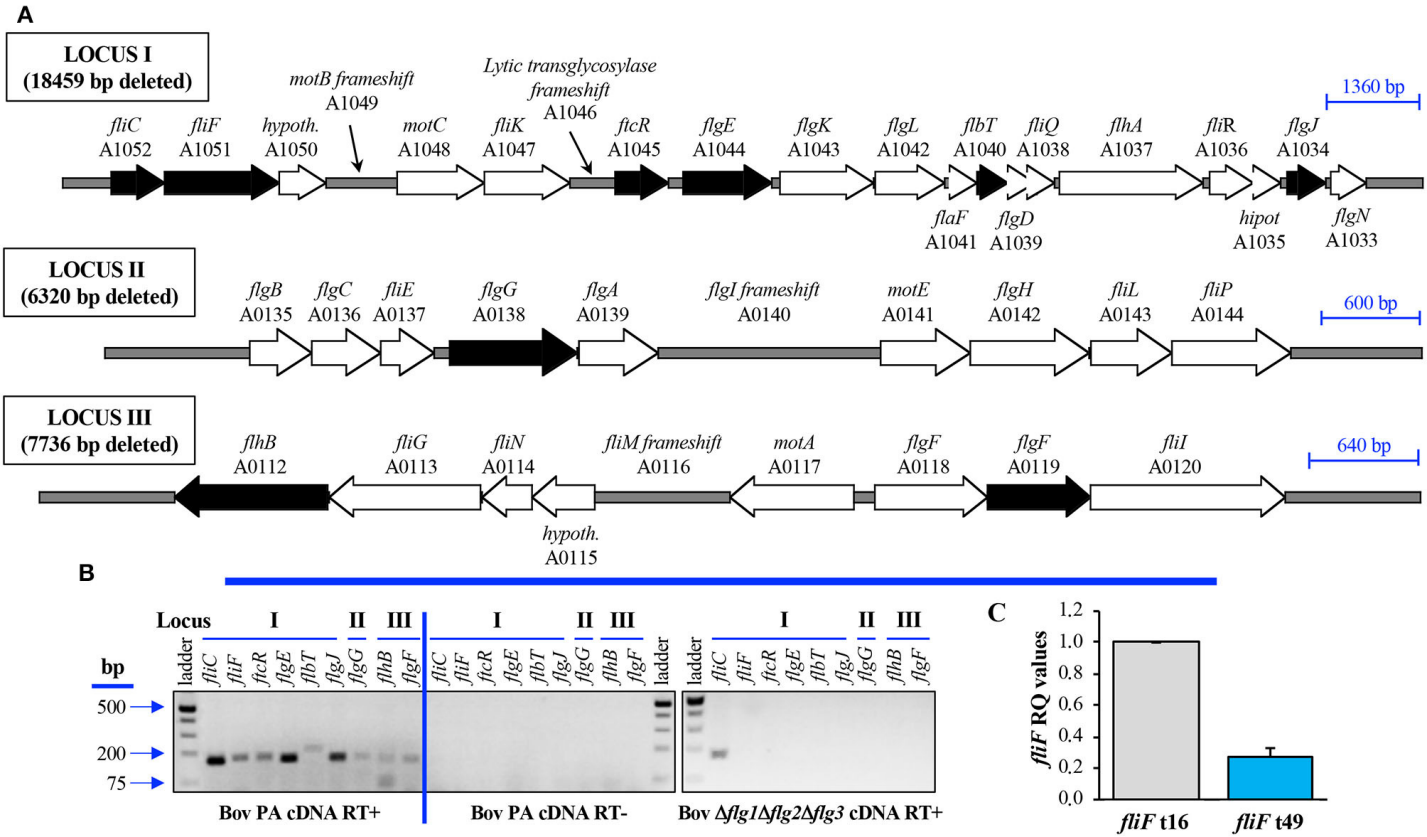
Genomic organization **(A)** and transcription **(B,C)** of the three main flagellar loci detected in the *B. ovis* genome. Flagellar genes targeted in transcription analysis with *B. ovis* PA strains **(B,C)** are represented with a black pattern **(A)**. Mutagenesis procedure deleted 99% of each locus **(A)** in flagellar mutants listed in Table 2. End-point RT-PCR **(B)** was performed with cDNA obtained by retrotranscription of RNA extracted at t16 from *B. ovis* PA or the Δ*flg1*Δ*flg2*Δ*flg3* triple mutant (RT+ reactions). Reactions of cDNA synthesis lacking RT were used as controls of DNA absence (RT- reactions). Amplification with *fliC* primers in the Δ*flg1*Δ*flg2*Δ*flg3* triple mutant **(B)** was expected, since both primers hybridize adjacent but externally to the deleted fragment (the deletion removes 80% of *fliC*). qRT-PCR **(C)** with *fliF* was performed with RNA extracted at t16 and t49 from *B. ovis* PA parental strain. Gene expression levels **(C)** were determined, with the StepOne^TM^ software v2.3, by the 2^−ΔΔCt^ method with the *16S* gene as internal reference and t16 results as control condition. Four biological replicates, with three technical replicates each, were analyzed and the results are expressed as means ± SD.

**Figure 2:**
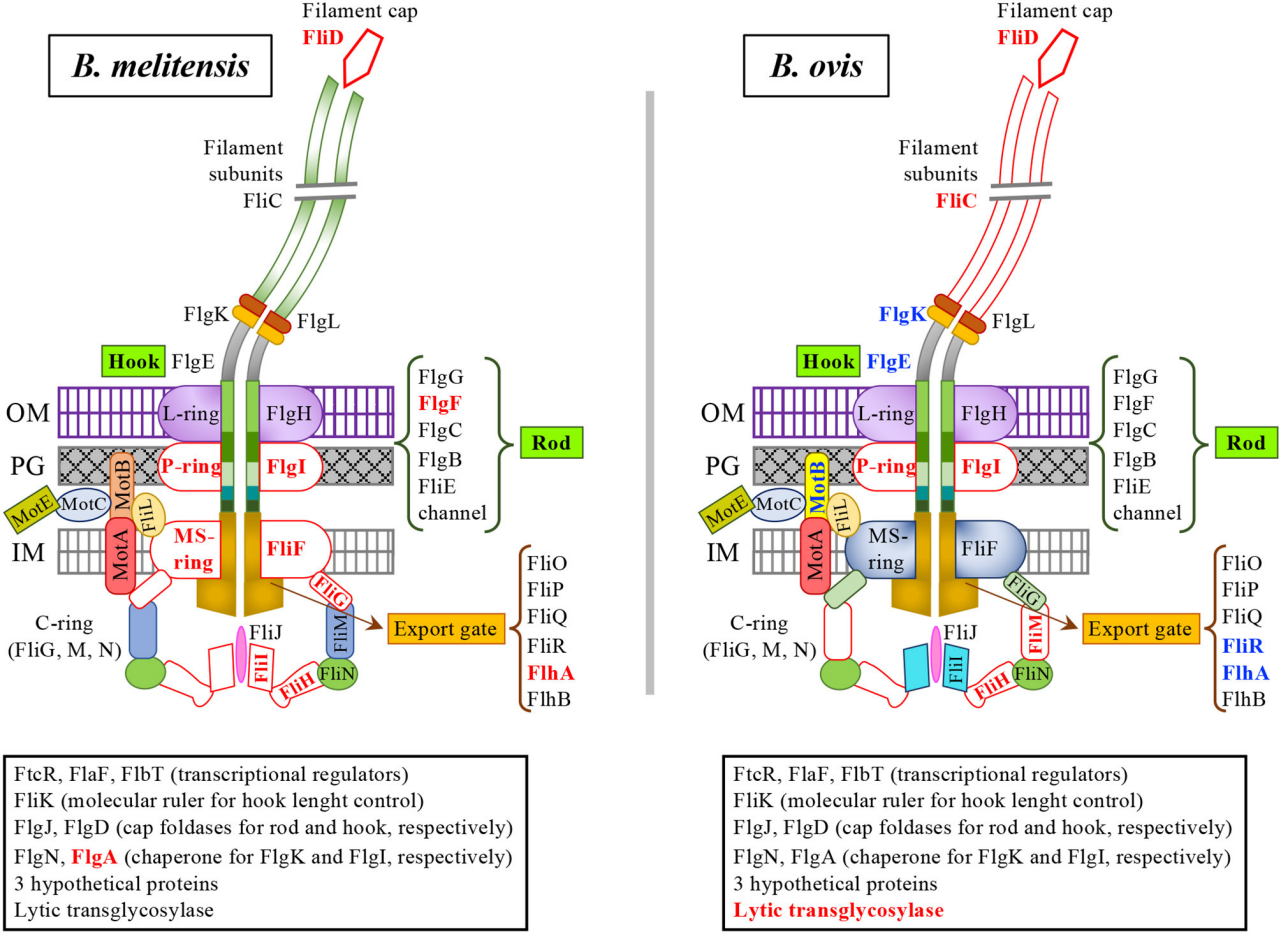
Schematic representation of the *B. melitensis* and *B. ovis* flagellum. The figure was elaborated according to the classic structure of the flagellum of Gram-negative bacteria (30–37) and the flagellar genes detected in the *Brucella* genomes (39 genes distributed in the three main flagellar loci of chromosome II and 4 genes found in chromosome I). Additional proteins detected in the flagellar loci intervening in flagellum biosynthesis or with unknown function are framed at the bottom of the figure. Flagellar proteins encoded by genes that, when compared to those of motile *Brucella* sp. B13-0095, contain frameshifts, premature stop codons or mutations affecting the start codon are written in red bold characters and represented by unfilled forms with red borders. This pattern has also been used for FliD and FliH, which have not been found in the *Brucella* genomes. *B. ovis* proteins with internal in-frame deletions in the encoding genes are written in blue bold characters. *B. ovis motB* in chromosome II contains a frameshift; the figure represents a MotB homolog encoded in chromosome I that is shorter than the *B. melitensis* ortholog due to an internal in-frame deletion in the encoding gene. C-ring (cytoplasmic ring), MS-ring membrane supramembrane ring, P-ring (peptidoglycan ring), L-ring (lipopolysaccharide ring), IM (inner membrane), PG (peptidoglycan), OM (outer membrane).

According to the annotated whole genome sequence of the *B. ovis* reference strain (29), the three flagellar loci of *B. ovis* (Figure 1A) present a similar organization to that described for *B. melitensis* 16M (11) and are also located in chromosome II. A search in the PATRIC genome of motile *Brucella* sp. B13-0095 revealed the presence of additional flagellar genes *motA, motB, fliJ*, and *fliO*, that were also detected in chromosome I of *B. melitensis* 16M and *B. ovis* 63/290 (Table 3). Hypothetical *motA* and *motB* genes were previously identified in locus III and locus I, respectively, of *Brucella* chromosome II (1, 11, 13) (Table 3) but the four hypothetical flagellar genes detected in chromosome I have not been reported before in studies targeting the *Brucella* flagellum (1, 11, 13). According to the flagellum structure described for Gram-negative bacteria (30–37), FliO would be part of the export gate (Figure 2) that extends from the membrane-supramembrane (MS ring) of the flagellum to the cytoplasm. FliJ, together with FliI and FliH, would constitute the ATPase complex (Figure 2) of the type III export machinery, although no gene potentially encoding FliH have been detected in the *Brucella* genomes. Similarly, *fliD* that encodes the filament cap protein in flagellated bacteria (Figure 2), has not been detected in the genus *Brucella*.

**Table 3:** Flagellar genes detected in the genomes of *Brucella* sp. B13-0095, *B. melitensis* 16M and *B. ovis* 63/290^a^.

**Gene identification in genome of** ***Brucella*** **spp**.			**Protein name (position in flagellum)^b^**	**Predicted subcellular localization (PSORTb)^c^**
**B13-0095**	**Bme 16M**	**Bov 63/290**		
**Chromosome II Locus I**			
BA060_07860	BMEII0150	BOV_A1052	FliC (filament)	Extracellular
BA060_07855	BMEII0151-52	BOV_A1051	FliF (MS-ring)	Cytoplasmic membrane
BA060_07850	BMEII0153	BOV_A1050	Hypothetical (unknown)	Cytoplasm
BA060_07845	BMEII0154	BOV_A1049	MotB (stator)	Periplasm/cytoplasmic memb.
BA060_07840	BMEII0155	BOV_A1048	MotC (stator)	Periplasm
BA060_07835	BMEII0156	BOV_A1047	FliK (hook molecular ruler)	Unknown
BA060_07830	BMEII0157	BOV_A1046	Lytic transglycosylase (unknown)	Not cytoplasm
BA060_07825	BMEII0158	BOV_A1045	FtcR (regulator)	Cytoplasm
BA060_07820	BMEII0159	BOV_A1044	FlgE (hook)	Extracellular
BA060_07815	BMEII0160	BOV_A1043	FlgK (hook-filament junction)	Outer membrane
BA060_07810	BMEII0161	BOV_A1042	FlgL (hook-filament junction)	Unknown
BA060_07805	BMEII0162	BOV_A1041	FlaF (regulator)	Unknown
BA060_07800	BMEII0163	BOV_A1040	FlbT (regulator)	Cytoplasm
BA060_07795	BMEII0164	BOV_A1039	FlgD (cap foldase for hook)	Extracellular
BA060_07790	BMEII0165	BOV_A1038	FliQ (export gate)	Cytoplasmic membrane
BA060_07785	BMEII0166-67	BOV_A1037	FlhA (export gate)	Cytoplasmic membrane
BA060_07780	BMEII0168	BOV_A1036	FliR (export gate)	Cytoplasmic membrane
BA060_07775	BMEII0169	BOV_A1035	Hypothetical (unknown)	Unknown
BA060_07770	BMEII0170	BOV_A1034	FlgJ (cap foldase for rod)	Unknown
BA060_07765	BMEII0171	BOV_A1033	FlgN (chaperone for FlgK)	Cytoplasm
**Chromosome II Locus II**			
BA060_08780	BMEII1089	BOV_A0135	FlgB (rod)	Unknown
BA060_08785	BMEII1088	BOV_A0136	FlgC (rod)	Periplasm
BA060_08790	BMEII1087	BOV_A0137	FliE (rod)	Unknown
BA060_08795	BMEII1086	BOV_A0138	FlgG (rod)	Periplasm
BA060_08800	BMEII1085	BOV_A0139	FlgA (chaperone for FlgI)	Cytoplasmic membrane
BA060_08805	BMEII1084	BOV_A0140	FlgI (P-ring)	Periplasm
BA060_08810	BMEII1083	BOV_A0141	MotE (chaperone for stator MotC)	Not cytoplasm
BA060_08815	BMEII1082	BOV_A0142	FlgH (L-ring)	Outer memb.
BA060_08820	BMEII1081	BOV_A0143	FliL (stator)	Cytoplasmic membrane
BA060_08825	BMEII1080	BOV_A0144	FliP (export gate)	Cytoplasmic membrane
**Chromosome II Locus III**			
BA060_08660	BMEII1114	BOV_A0112	FlhB (export gate)	Cytoplasmic membrane
BA060_08665	BMEII1113	BOV_A0113	FliG (C-ring)	Cytoplasm
BA060_08670	BMEII1112	BOV_A0114	FliN (C-ring)	Cytoplasmic membrane
BA060_08675	BMEII1111	BOV_A0115	Hypothetical (unknown)	Not cytoplasm
BA060_08680	BMEII1110	BOV_A0116	FliM (C-ring)	Cytoplasm
BA060_08685	BMEII1109	BOV_A0117	MotA (stator)	Cytoplasmic membrane
BA060_08690	BMEII1108	BOV_A0118	DUF1217 domain protein (unknown)	Unknown
BA060_08695	BMEII1107	BOV_A0119	FlgF (rod)	Periplasm
BA060_08700	BMEII1106-05	BOV_A0120	FliI (ATPase complex)	Cytoplasm
**Chromosome I genes**			
BA060_12400	BMEI0948	BOV_1003	FliO (export gate)	Unknown
BA060_11245	BMEI0422	BOV_1543	FliJ (ATPase complex)	Cytoplasm
BA060_01660	BMEI0325	BOV_1655	MotA (stator)	Cytoplasmic membrane
BA060_01665	BMEI0324	BOV_1656	MotB (stator)	Cytoplasmic membrane

a *Brucella sp. B13-0095 is a motile strain isolated from a Pac-Man frog (Ceratophrys ornata) (13), B. melitensis 16M is the type strain of the genus and is able to build a sheathed flagellum in particular culture conditions and B. ovis 63/290 is the B. ovis type strain. Red lettering indicates premature stop codons or frameshifts and blue lettering indicates internal in-frame deletions, when compared to the genes of Brucella sp. B13-0095*.

b *Protein identification according to the annotation in the B. ovis 63/290 or Brucella sp. B13-0095 (for chromosome I genes) genomes. Position in flagellum as shown in Figure 2 according to the general structure described for flagella of Gram-negative bacteria (30–37)*.

c *PSORTb v3.0.2 predicts subcellular localization of bacterial proteins (https://www.psort.org/psortb/). The analysis was performed with the proteins of B. ovis 63/290 or Brucella sp. B13-0095 (for B. ovis 63/290 frameshifted proteins)*.

When compared to the genome of motile flagellated *Brucella* sp. B13-0095 isolated from a Pac-Man frog (13), *B. melitensis* 16M and *B. ovis* exhibited a different pattern of defective genes (Table 3, Figure 2) with the characteristics listed in Table 4. In the *B. melitensis* 16M genome, six flagellar genes with premature stop codons or frameshifts have been detected (Tables 3, 4). Additionally, the hypothetical start codon and ribosome binding site of the *B. melitensis* 16M *flgA* gene -coding for a putative chaperone for the FlgI P-ring protein (whose gene is frameshifted when compared to *Brucella* sp. B13-0095 *flgI*)- are lost due to a deletion of 18 nt. In *B. ovis* 63/290, five flagellar genes contain internal in-frame deletions shortening the encoded protein (Table 3, blue lettering, and Table 4) and six additional genes (Figure 1A, Table 3, red lettering, and Table 4) contain premature stop codons or frameshifts. However, flagellin *fliC* gene is not annotated as pseudogene in the whole genome sequence of *B. ovis*. Since in this work we have used virulent *B. ovis* PA, the possibility of some differences with *B. ovis* 63/290 cannot be discarded. However, previous genomic studies with *B. ovis* PA provided the same results as *B. ovis* 63/290 (18, 19, 38, 39) and all sequences we have determined in this work for the construction of flagellar mutants (including internal sequences not reported here) were identical to those of *B. ovis* 63/290.

**Table 4:** Defective flagellar genes in *B. melitensis* 16M and *B. ovis* 63/290^a^.

**Gene identification in the genome of** ***Brucella*** **spp**.		**Protein**	**Relevant gene defect(s) when compared to motile *Brucella* sp. B13-0095^b^**
***B. melitensis*** **16M**			
Locus I	BMEII0151-52	FliF	1 nt substitution leading to premature stop codon
	BMEII0166-67	FlhA	1 nt substitution leading to premature stop codon
Locus II	BMEII1085	FlgA	18 nt deletion involving start codon and probable ribosome binding site
	BMEII1084	FlgI	First 6 nt differ affecting start codon 1 nt deletion leading to frameshift
Locus III	BMEII1113	FliG	Internal 83 nt deletion with frameshift
	BMEII1107	FlgF	1 nt deletion leading to frameshift
	BMEII1106-05	FliI	1 nt insertion leading to frameshift
***B. ovis*** **63/290**			
Locus I	BOV_A1052	FliC	Internal 48 nt in-frame deletion C-terminal 203 nt deletion leading to frameshift and affecting the intergenic FliC-FliF region
	BOV_A1049	MotB	35 nt deletion leading to frameshift
	BOV_A1046	Lytic transglyc.	1 nt deletion leading to premature stop (frameshift) codon 71 nt deletion
	BOV_A1044	FlgE	Internal 57 nt in-frame deletion
	BOV_A1043	FlgK	Internal 42 nt in-frame deletion Internal 18 nt in-frame deletion
	BOV_A1037	FlhA	Internal 36 nt in-frame deletion
	BOV_A1036	FliR	Internal 48 nt in-frame deletion
Locus II	BOV_A0140	FlgI	First 6 nt differ affecting start codon 1 nt deletion leading to frameshift
Locus III	BOV_A0116	FliM	1 nt substitution leading to premature stop codon
Chrom. I	BOV_1655	MotA	Internal 31 nt deletion leading to frameshift
	BOV_1656	MotB	Internal 87 nt in-frame deletion

a *Compared to the genes of motile Brucella sp. B13-0095 isolated from a Pac-Man frog (Ceratophrys ornata) (13)*.

b *Relevant defects included in the table are nucleotide deletions, insertions or substitution leading to in-frame deletions, frameshift or premature stop codons. Nucleotide substituions that do not introduce premature stop codons are not considered as relevant defects*.

To evaluate whether flagellar loci are transcribed in *B. ovis* PA, end-point RT-PCR was performed with RNA extracted at the exponential phase of growth and primers targeting nine genes distributed in the three main loci. Transcription of all evaluated genes was detected in the parental strain *B. ovis* PA (Figure 1B), while no amplification was observed with cDNA obtained from the Δ*flg1*Δ*flg2*Δ*flg3* mutant, except for *fliC* (Figure 1B). This exception was expected because the selected primers for RT-PCR amplify a 164 nt fragment of the 5′-end of *fliC* that is externally bordering the deleted DNA fragment of locus I (deletion removes 99% of locus I, which includes 80% of *fliC*). Studies of relative expression in TSB-YE-HS liquid medium of the locus I *fliF* gene (performed by qRT-PCR using *16S* as internal reference gene) showed that *fliF* is down regulated (with about 3.5-fold reduction) in the stationary growth phase (t49) when compared to the exponential growth phase (t16) (Figure 1C).

### Construction, Growth, and OM-Related Properties of *B. ovis* PA Flagellar Mutants

Three initial *B. ovis* PA mutants were constructed (*B. ovis* Δ*flg1*, Δ*flg2*, and Δ*flg3*), each with one of the three main flagellar loci deleted (locus I, II, and III, respectively). Despite the high size of the deleted fragment, mainly in *B. ovis* Δ*flg1* (about 18 kb deleted), no difficulties were found to obtain the three mutants. Similarly, double and triple mutants combining the deletion of two or the three flagellar loci, were obtained (32.5 kb deleted in triple mutants). All possible combinations of double and triple mutants were obtained in order to set out a panel of mutants that could be analyzed in case of discovering a differential behavior in one mutant and thus minimize the risk of attributing differences caused by other undesired mutations to the absence of flagellar loci.

No differences in growth in solid medium were observed in single, double or triple mutants that showed similar CFU/ml values for bacterial suspensions of OD_600_ = 0.2 than the parental strain (data not shown). Equivalent results among strains were also observed in TSB-YE-HS liquid medium with a similar evolution of OD_600_ values and CFU/ml with time (Supplementary Figure 1). In the autoagglutination assay no differences were found among strains since, as expected for the parental *B. ovis* PA strain (26), all of them remained in suspension (Supplementary Figure 2). According to these results, only three triple mutants (*B. ovis* Δ*flg1*Δ*flg2*Δ*flg3*, Δ*flg2*Δ*flg1*Δflg3 and Δ*flg3*Δ*flg2*Δ*flg1*, which have the three flagellar loci deleted in a different order) were initially selected for the remaining studies. The other mutants would only be analyzed if differences were found with the triple mutants.

Properties related to the OM, and that have also been related to survival in the host, were evaluated in the selected triple mutants in comparison with *B. ovis* PA. Diameters of growth inhibition halos obtained by exposure to H_2_O_2_, sodium deoxycholate or polymyxin B did not show relevant differences between the three triple flagellar mutants and the parental strain (Supplementary Figure 3).

### Virulence of *B. ovis* PA Flagellar Mutants

**Figure 3:**
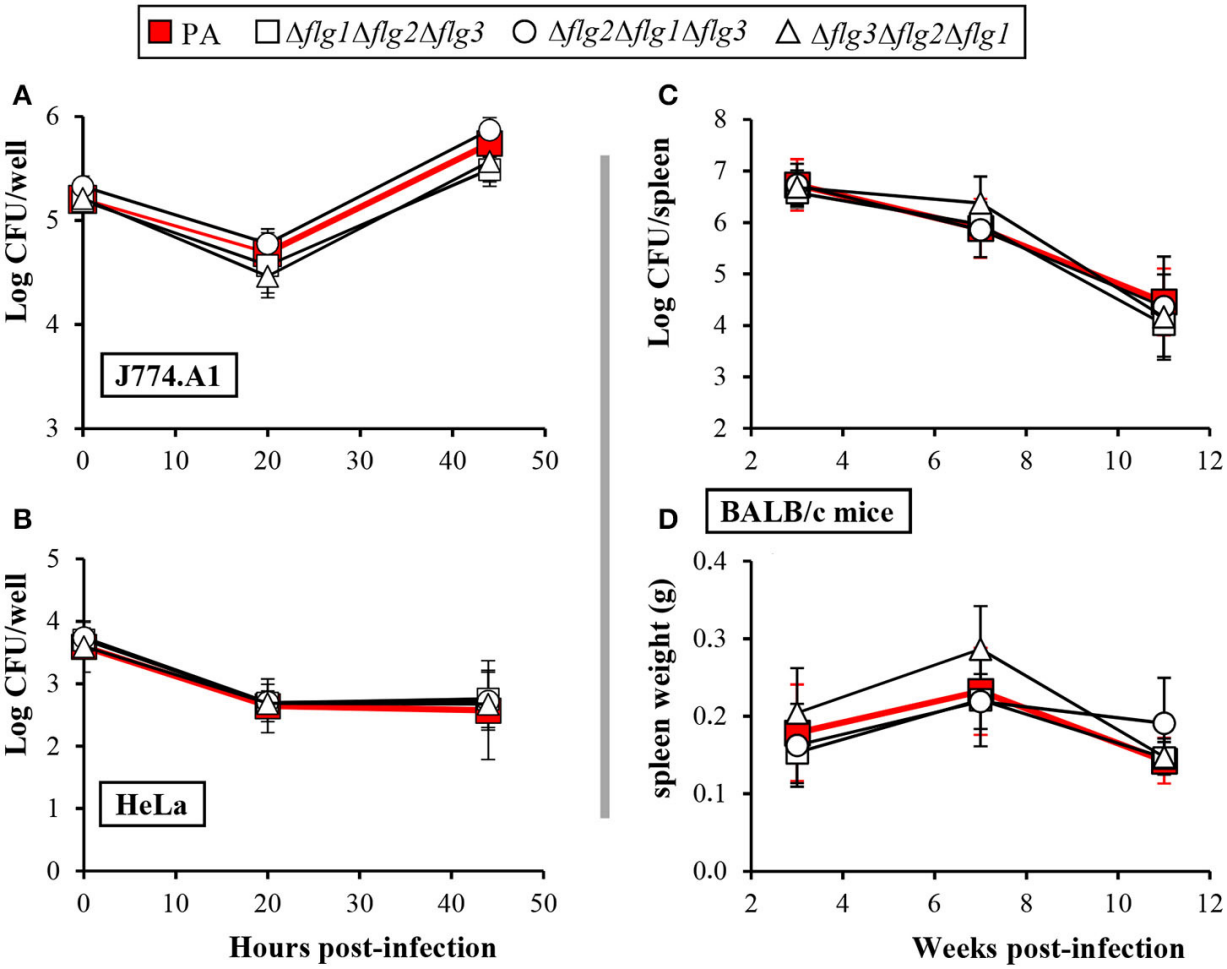
Virulence of *B. ovis* PA triple mutants in flagellar loci. Virulence assays were performed in J774.A1 **(A)** and HeLa **(B)** cell lines and in BALB/c mice **(C,D)**. Parental *B. ovis* PA was included in the experiments for comparisons. In cell assays **(A,B)**, the results are expressed as the means ± SD (*n* = 3) of the log CFU/well at each time point. Virulence in mice is expressed as the means ± SD (*n* = 5) of the log CFU/spleen at each time point **(C)**. Spleen weight in the same mice is also represented **(D)**.

Since *B. ovis* is an intracellular pathogen (6, 17, 19, 26, 40), the behavior in J774.A1 and HeLa cells of the triple mutants was evaluated in comparison to that of the parental strain. Removal of the three main flagellar loci in *B. ovis* PA did not affect the internalization of the bacterium or its intracellular evolution (Figures 3A,B). These results were somehow expected since flagellar mutants of *B. melitensis* 16M did not show an altered intracellular pattern (11), although it must be taken into account that each one of these mutants was only defective in one single gene.

On the contrary, while single gene mutants of *B. melitensis* 16M were unable to establish a chronic phase of infection in mice (11), the three triple mutants of *B. ovis* PA analyzed, lacking the 32.5 kb of the three main flagellar loci, did not show attenuation in BALB/c mice (Figure 3). Thus, both the splenic bacterial counts (Figure 3C) and the spleen weight (Figure 3D) of the flagellar mutants showed the same temporal evolution than those observed with the parental strain.

## Discussion

Since the classical *Brucella* species lack motility (41) and display random patterns of pseudogenization (1), it is tempting to hypothesize that flagellar loci, which are conserved in the genus *Brucella*, are remnants of an environmental ancestor that no longer required motility after the evolutive adaptation to the animal host and to an intracellular lifestyle. However, the detection of a flagellum in *B. melitensis* 16M and the involvement of flagellar genes in its virulence in mice (11, 42, 43) and probably in goats (44) raises a new perspective. Moreover, although the relevance for virulence remains unexplored, the recently reported motility of atypical *Brucella* strains (1, 2, 12–14) and their ability, at least for amphibian isolates, to build a polar flagellum (1) also encourages additional studies to elucidate the function of flagellar genes in the genus *Brucella*. In this work, we have selected a virulent *B. ovis* strain to construct and characterize a panel of mutants in flagellar loci with a main focus in virulence.

Although the profile of defective flagellar genes detected in *B. ovis* (Figure 2, Tables 3, 4) makes the assembly of a flagellum unlikely, *B. melitensis* 16M also contains defective genes (Figure 2, Tables 3, 4) that would not be compatible with the synthesis of a flagellum. Therefore, it is probable that either the modified proteins are functional or *B. melitensis* 16M is able to synthetize at least some whole-length molecules by suppression of the stop codons (i.e., *fliF* and *flhA*) (11) and compensation of DNA frameshifts (i.e., *flgI, fliG, flgF*, and *fliI*) by transcription slippage or ribosomal frameshift (45). Accordingly, the defects observed in some *B. ovis* flagellar genes do not necessarily imply the impossibility of assembling a complete or partial flagellar structure that could contribute to virulence.

We have detected that *B. ovis* PA is able to transcribe flagellar genes located in the three main loci and that, as described for *B. melitensis* 16M (11), the transcription level is higher in the exponential growth phase, at least for *fliF* (Figures 1B,C). To our knowledge, no other studies have evaluated expression of flagellar genes in *B. ovis*, either in culture medium or inside phagocytes, and the sole study that analyzed the intracellular transcriptome of *B. ovis* did not report upregulation or downregulation of flagellar genes (46). However, expression of *B. ovis* flagellar genes in an intracellular environment, as it has been reported for *B. melitensis* 16M (11), cannot be discarded. Although this aspect would merit further attention, our results clearly demonstrate that the entire three main flagellar loci of *B. ovis* PA (accounting for 39 genes) are dispensable for all properties evaluated, including intracellular survival and virulence in the mouse model (Figure 3 and Supplementary Material). Therefore, most likely *B. ovis* does not build a flagellum or, if it does, it would not be required for the establishment of infection.

Since the mechanism/s responsible for the contribution of flagellum to virulence of *B. melitensis* 16M has not been elucidated, it is difficult to hypothesize about how the presumed absence of flagellum in *B. ovis* has influenced the host-pathogen interaction. Flagella may be involved in the four main stages of the infectious process of bacterial pathogens (47): (i) reaching the host or target site (ii) colonization and invasion, (iii) maintenance and replication, and (iv) dispersal to new hosts. The absence of motility (at least *in vitro*) and of chemotactic systems would exclude the first role in the classical *Brucella* species. Role in adhesion and invasion of host cells (at least in cell cultures) is also unlikely, since *B. melitensis* flagellar mutants show no internalization defects in HeLa cells or in bovine peritoneal macrophages (11). The same cellular models also revealed that, even though the *fliF* promoter is induced intracellularly, the flagellum is not required for intracellular replication of *B. melitensis* 16M (11). Additionally, attenuation in mice of *B. melitensis* 16M flagellar mutants was not detected at 1W p.i., but only at later time points (11, 43). However, *in vivo* mouse imaging technology showed that a luminescent *B. melitensis* 16M flagellar mutant, lacking four genes coding for rod proteins, had a limited ability to disseminate from the point of intraperitoneal inoculation (48). This impaired dissemination might be related to the impossibility of *B. melitensis* 16M flagellar mutants to establish a chronic infection in mice (11). But also, if this behavior were reproduced in the natural host, the flagellum could be responsible, at least in part, for the tropism of *B. melitensis* 16M by the placenta. This statement would be in accordance with the fact that *B. melitensis* infections frequently induces abortions (21) while *B. ovis* (that share with *B. melitensis* the preference for the ovine host) exhibits a marked tropism by the male genital tract and is seldom associated to abortions (20) despite its ability to internalize and replicate in trophoblasts (6). Moreover, a contribution of the flagellum to the zoonotic potential of *B. melitensis*, which is the highest of the genus, should also be considered. More studies involving flagellar genes in other *Brucella* species associated with abortions in their preferred hosts or able to infect humans would help to clarify these points.

The exacerbated virulence pattern, accompanied by histological damage in spleen, that was observed in BALB/c mice with a non-polar Δ*fliC* mutant of *B. melitensis* 16M constitutes an additional remarkable observation regarding flagellar genes (43). It was proposed that FliC flagellin of *B. melitensis* 16M triggers the innate immune response and that a tight regulation of flagellar expression in this strain is part of the stealthy strategy that allows to maintain a persistent infection without severely damaging host tissues (43). Some other evidences point to the requirement for a finely tuned regulation of flagellar genes in *B. melitensis* 16M to establish a persistent infection: (i) the large number of reported direct or indirect regulators of flagellar gene expression: FtcR, FlbT, and FlaF, which are encoded in flagellar locus I (Figure 1A), or VjbR, BlxR, RpoE1, BpdA, and YbeY (48–54), (ii) the results obtained in an *in vivo* model simulating the onset of *B. melitensis* 16M infection in cattle (first 4 h) showing that the three main flagellar loci were repressed while transcription of the *rpoE1* repressor gene was activated (55), and (iii) the flagellum is sheathed by an extension of the outer membrane ending by a club-like structure that has been suggested to contribute to the assembly of FliC flagellin subunits (42) in the absence of the filament cap protein FliD (Figure 2) that has not been detected in the *Brucella* genomes; since the *Brucella* outer membrane is considered as part of its stealthy strategy to establish persistent infections (56), the flagellar sheath could contribute to limit FliC presentation to the immune system.

In the case of *B. ovis* PA and even if a flagellum is not assembled, flagellin is likely to be synthetized, since this strain is able to transcribe *fliC* (Figure 1B) and *B. melitensis* 16M synthesizes FliC even in the absence of deeper flagellar structural proteins such as FliF (basal body protein) or FlgE (hook protein) (53). Therefore, flagellin might be translocated to the cytoplasm of the host cell through the type-IV secretion system (encoded by the *virB* operon), as it has been suggested for *B. melitensis* 16M, and induce an innate immune response mediated by cytosolic NCRC4 receptors (43). However, even if *B. ovis* PA produces flagellin and is able to translocate it into the host cell cytoplasm, it would not be relevant in the induction of a detrimental immune response for the bacterium because flagellar mutants behave as the parental strain (Figure 3). This fact could be related with the differences detected in the C-terminal residues of FliC in *B. ovis* when compared to the protein of *B. melitensis* (Tables 3, 4). Both N- and C-terminal domains are involved in the self-polymerization of flagellin subunits (35, 57), but C-end residues have also been proposed as targets for the innate immune response sensed by NLRC4 receptors (43). On the other hand, presentation of assembled flagellin subunits in a surface-exposed flagellar structure could be essential to induce the immune response (and/or to interact with its effectors), and the pattern of defective flagellar genes in *B. ovis* would not allow this requirement.

Another intriguing observation regarding the *B. melitensis* 16M flagellum is the contrast between the exacerbated virulence in mice of the Δ*fliC* mutant (unable to synthetize flagellin and therefore the filament of the flagellum) and the attenuation of the Δ*fliF* mutant (unable to synthetize the MS-ring proteins) (43). This characteristic suggests that the flagellar export channel of *B. melitensis* 16M could also be used for the transport of molecules required to maintain a chronic infection in the host. Whether the Type III machinery involved in the specialized export of the flagellar subunits (32, 33) contributes to the export of other molecules in *B. melitensis* 16M that might participate in virulence has not been elucidated. If this were the case and considering the full virulence of flagellar mutants (Figure 3), this possibility does not seem to occur in *B. ovis* either by a naturally defective channel or by the absence of the hypothetical virulence determinants.

The demonstration that flagellar genes are dispensable for *B. ovis* virulence in mice (Figure 3) constitutes a new particular characteristic of this rough species to add to the previously reported differences with other brucellae (16–19). To build a profile of differential characteristics for each *Brucella* species would contribute to decipher the mechanisms underlaying the differences of pathogenicity and host preference that exist between the classical *Brucella* species despite their high similarity at the DNA level. However, although the mouse model usually mimics the results obtained in the natural host for attenuated mutants, it has limitations (58, 59), and *Brucella* mutants exhibiting whole virulence in the mouse model but attenuated in the natural host have been reported (60). Therefore, although unlikely, a role of *B. ovis* flagellar genes in the natural host cannot be completely discarded. More studies in other *Brucella* species, including abortifacient and zoonotic *Brucella* species and the recently isolated motile strains, would help to clarify the relevance of flagellar genes in the genus *Brucella*.

## Data Availability Statement

All datasets generated for this study are included in the article/Supplementary Material.

## Ethics Statement

Mice experiments were designed according to the Spanish and European legislation for research with animals (RD 53/2013 and directive 86/609/EEC). Microbiological procedures and experimentation with mice were approved by the Biosecurity and Bioethics Committees of the University of Salamanca and certified by the competent authority of Junta de Castilla León, Spain.

## Author Contributions

RS-M and NV conceived the study and wrote the manuscript. RS-M, CT, and NV participated in the experimental work, the discussion of the results, and the revision of the manuscript. All authors read and approved the final version of the manuscript.

## Conflict of Interest

The authors declare that the research was conducted in the absence of any commercial or financial relationships that could be construed as a potential conflict of interest.
