# Supplementary figures and images for: The Three Flagellar Loci of Brucella ovis PA Are Dispensable for Virulence in Cellular Models and Mice
Source: Front Vet Sci. 2020 Jul 31;7:441. doi: 10.3389/fvets.2020.00441 (PMC7410920; doi:10.3389/fvets.2020.00441)

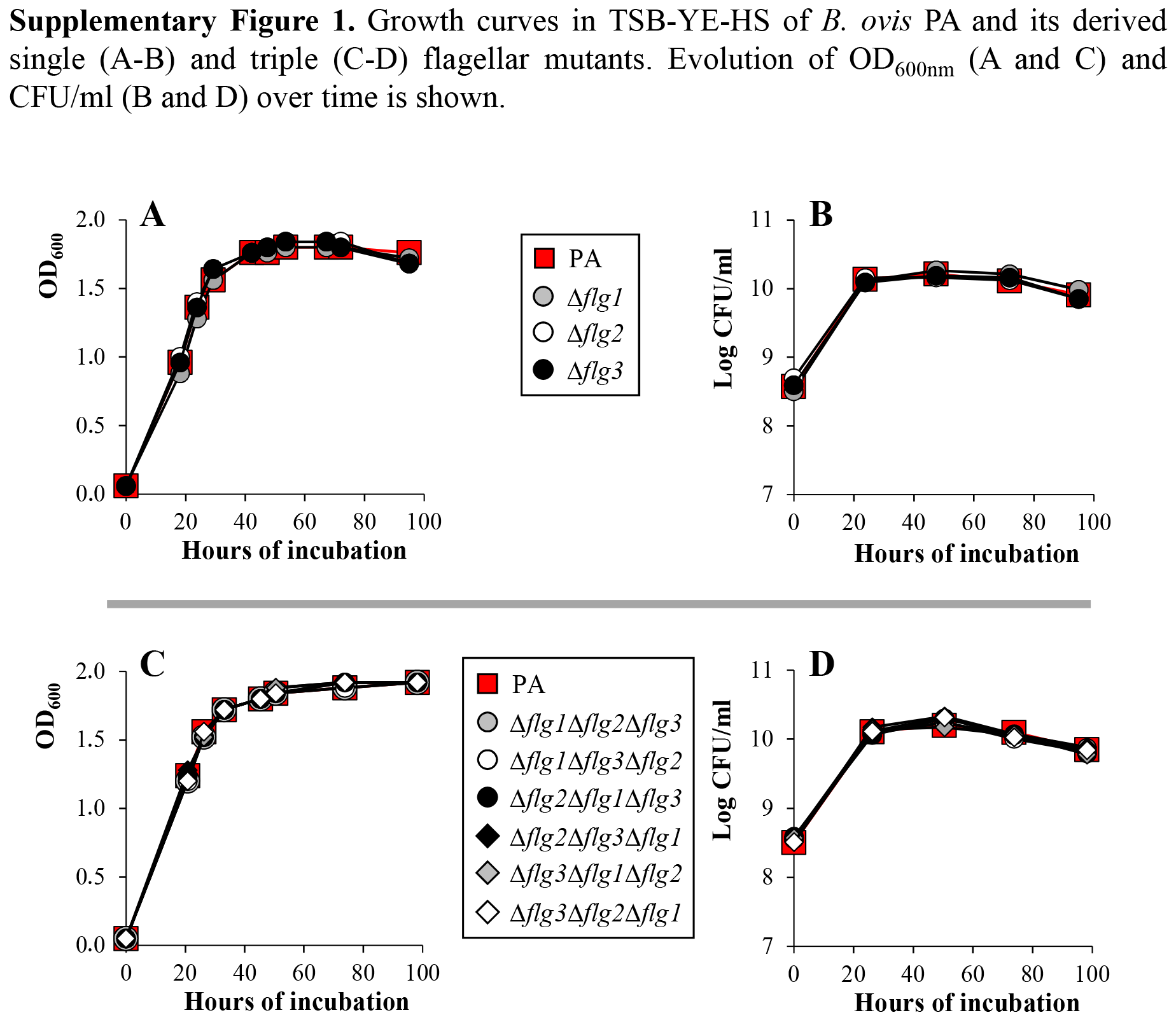

Supplement: Supplementary file 1 [file Image_1.TIF]

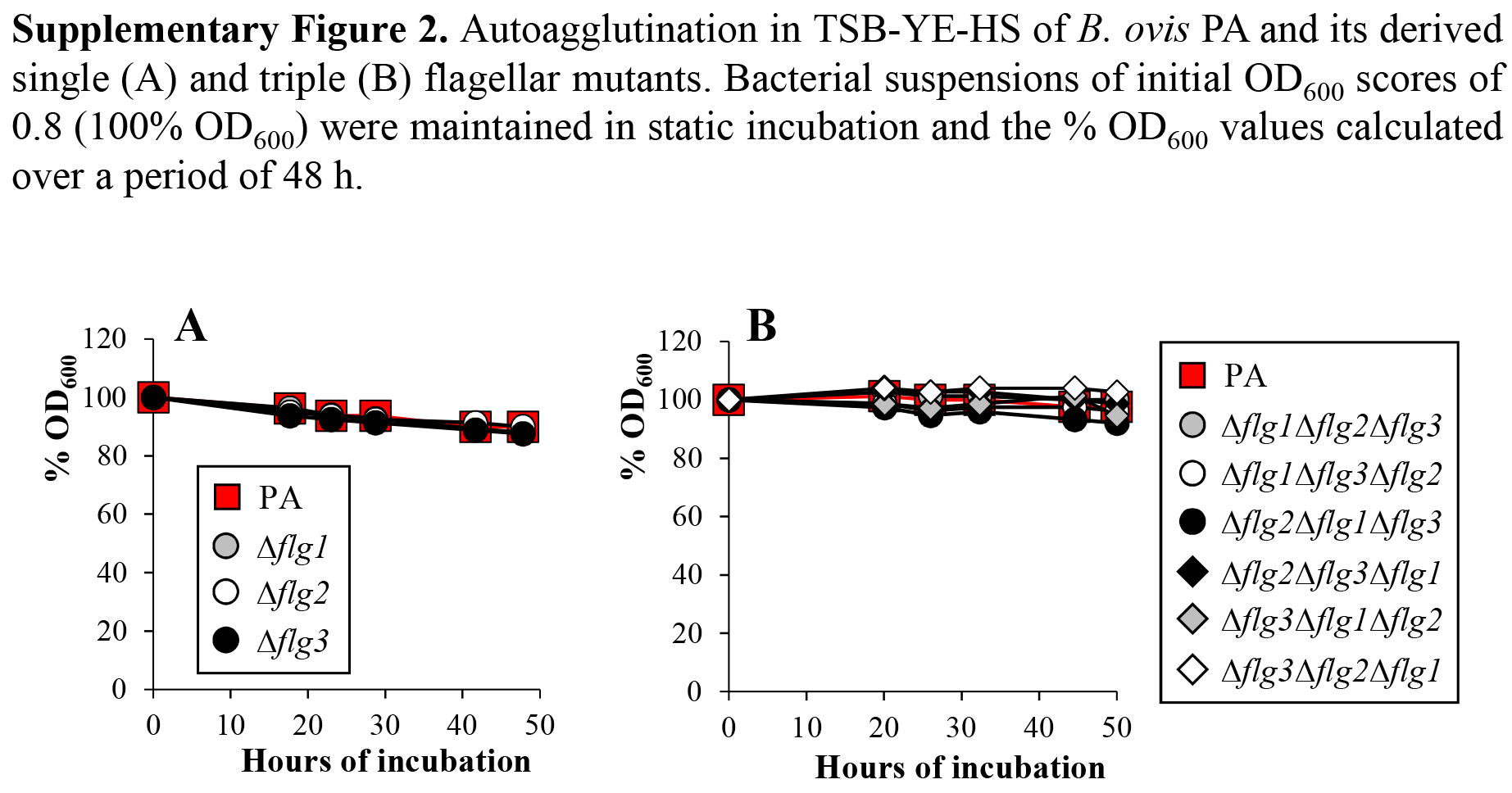

Supplement: Supplementary file 2 [file Image_2.TIF]

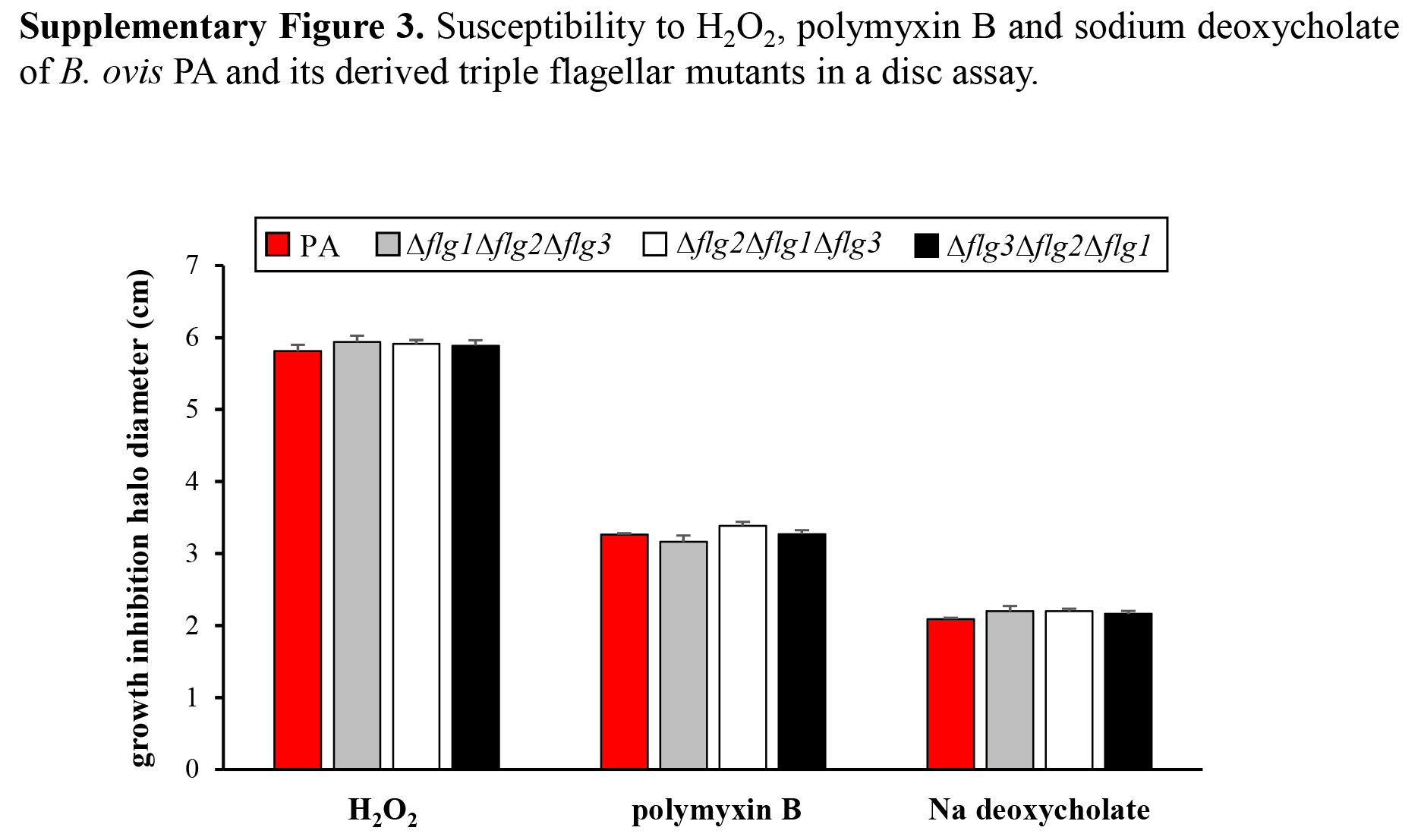

Supplement: Supplementary file 3 [file Image_3.TIF]
